# Effect of channel length on the electrical response of carbon nanotube field-effect transistors to deoxyribonucleic acid hybridization

**DOI:** 10.3762/bjnano.5.217

**Published:** 2014-11-12

**Authors:** Hari Krishna Salila Vijayalal Mohan, Jianing An, Yani Zhang, Chee How Wong, Lianxi Zheng

**Affiliations:** 1 School of Mechanical and Aerospace Engineering, Nanyang Technological University, 50 Nanyang Avenue, 639798, Singapore; 2 Temasek Laboratories, Nanyang Technological University, 50 Nanyang Avenue, 639798, Singapore; 3 Science and Technology on Thermostructural Composite Materials Laboratory, Northwestern Polytechnical University, 710072, Xi'an, PR China,; 4 Department of Mechanical Engineering, Khalifa University of Science, Technology & Research (KUSTAR), P.O. Box 127788, Abu Dhabi, United Arab Emirates

**Keywords:** biosensor, carbon nanotubes, channel length, field-effect transistor, hybridization, mobility, nucleic acid

## Abstract

A single-walled carbon nanotube (SWCNT) in a field-effect transistor (FET) configuration provides an ideal electronic path for label-free detection of nucleic acid hybridization. The simultaneous influence of more than one response mechanism in hybridization detection causes a variation in electrical parameters such as conductance, transconductance, threshold voltage and hysteresis gap. The channel length (*L*) dependence of each of these parameters necessitates the need to include them when interpreting the effect of *L* on the response to hybridization. Using the definitions of intrinsic effective mobility (µ_e_) and device field-effect mobility (µ_f_), two new parameters were defined to interpret the effect of *L* on the FET response to hybridization. Our results indicate that FETs with ≈300 µm long SWCNT exhibited the most appreciable response to hybridization, which complied with the variation trend in response to the newly defined parameters.

## Introduction

Detection of nucleic acids such as deoxyribonucleic acid (DNA) or ribonucleic acid (RNA) is an important issue in the field of biomedical and life science research [1–2]. Currently, the detection methods for nucleic acids include northern blotting analysis [3], in situ hybridization [4], real-time fluorescence quantitative polymerase chain reaction (RT-PCR) [5] and microarrays [6]. These detection methods suffer from several limitations, such as: a large sample volume requirement, complex data analysis, requirement of fluorescent- or radio-labelling, low sensitivity and high cost in addition to requiring multiple steps. The need for a simple, robust, label-free ultrasensitive technique is needed to overcome those shortcomings. Therefore, attention has shifted towards the use of nanowires and nanotubes for cost-effective, sequence-selective and label-free electrical detection without the use of the PCR for rapid measurement [7].

Semiconducting single-walled carbon nanotubes (SWCNTs) are excellent one-dimensional electronic materials, and their further development has been encouraged in high frequency applications [8–9], chemical sensing [10–11] and biosensing [12–13]. Recently, SWCNTs have been demonstrated as one of the best biosensors for a number of reasons: i) their diameter (several nanometers) is comparable to the size of single biomolecules, and to the electrostatic screening length in physiological solutions, which offers high sensitivity because of their large specific area; ii) their tubular structure allows fabrication of ultrasensitive, single nanotube-based devices; and iii) their excellent chemical stability favors the use of various functionalization schemes to improve the specificity and selectivity during sensing [14–15]. For instance, SWCNTs used in a field-effect transistor (FET) configuration are capable of electronically detecting nucleic acids because of their ability to respond to induced surface charges, which in turn, modulates their electrical transport [16–17]. However, most of the previous FET-based investigations for DNA–DNA hybridization detection predominantly used short individual nanotubes or random networks, which revealed Schottky barrier modification as their dominant sensing mechanism in a dry back-gated configuration [18–19]. A major issue with the Schottky barrier dependent mechanism is distinguishing hybridization from other biological events in the presence of CNT–metal contact electrodes during detection. These events increase the risk of a false signal arising from contaminants during signal analysis, which might affect the sensitivity and specificity of target detection. The efficacy of SWCNT-based FETs for hybridization detection depends on the number of hybridizations occurring on the SWCNT surface; therefore, it is important to understand the influence of the channel length on hybridization detection. One proposed method to confine hybridization events on the channel surface and to reduce the influence of contacts is the use of long CNTs. Thus the signal response is a consequence of the alteration in the intrinsic electronic property of the SWCNT alone. Earlier studies suggest that long SWCNTs, particularly those in the range of a few hundred micrometers, have a large coverage area, excellent normalized conductance, and extraordinary mobility at room temperature [20–21]. These properties open avenues for detecting hybridization occurring on the nanotube surface. However, with increasing channel length, the large channel resistance could be a limiting factor in the detection sensitivity. In particular, the FET electrical parameters such as conductance, transconductance, threshold voltage and hysteresis gap extracted from the current–voltage characteristics, which are indicators of the various contributing FET biosensing mechanisms [19], vary with channel length. A change in more than one of these electrical parameters after hybridization indicates the influence of multiple FET mechanisms. Therefore, it becomes essential to take into consideration the multiple parameters when interpreting the effect of channel length on the FET response.

In this paper, we show the controlled procedure of a SWCNT-based, FET system that allows for detection of DNA hybridization. In this system, FETs with short, long and ultra-long channel lengths were fabricated, and individual SWCNTs were functionalized. Furthermore, we investigated the effect of channel length on the response of the SWCNT-based FET to hybridization. In a separate experiment, certain parameters were defined using the intrinsic effective mobility and device field-effect mobility, which define the change in the aforementioned electrical parameters caused by hybridization, in order to interpret the variation in the response with channel length.

## Experimental

### SWCNT growth

Individual, long SWCNTs were grown on n-type Si capped by 1 µm thick, thermally grown SiO_2_. A gas-flow-guided ethanol chemical vapor deposition (CVD) process at 950 ºC was utilized to grow SWCNTs, in which 0.01 M FeCl_3_ ethanol solution was used as the catalytic precursor similar to our previous works [22–23].

### Fabrication of SWCNT-based FETs

We prepared three types of SWCNT-based FETs with different channel lengths, *L*, namely: (i) short channels (*L* = 6 µm), (ii) long channels (*L* = 300 µm), and (iii) ultra-long channels (*L* = 1500 µm). To obtain the short channel FETs, source–drain electrodes were patterned onto the substrates containing SWCNTs using standard photolithography (AZ7220, positive photoresist) at 25 ºC, followed by deposition of Ti (5 nm)/Au (50 nm) through electron beam evaporation (≈8 × 10^−4^ Pa) and a lift off process using acetone. The long and ultra-long channel FETs were fabricated by shadow mask-facilitated, electron beam evaporation using the same parameters. These were deposited onto the Si/SiO_2_ substrates containing the CVD-grown SWCNTs. The 1 µm thick SiO_2_ acted as the dielectric gate. Selective photoresist capping using photolithography was applied to cover the CNT–metal junction which resulted in devices with only the channel exposed. Only devices with a single CNT as a channel were considered for the detection studies. Specifically, only semiconducting FETs were used for the study.

SEM (Jeol, JSM-7600F) was used to verify the existence of the CNTs between the electrodes. AFM (Asylum Research, Cypher AFM) in tapping mode was used to obtain the height profile, which gives the diameter of the CNT. To confirm the semiconducting nature of the SWCNT devices, Raman spectroscopy was performed using a Raman spectrometer (Renishaw, inVia Raman Microscope). For the Raman measurements, the laser power was kept below 1 mW and an excitation wavelength of 633 nm was used. The radial breathing mode (RBM) and G-band were used for verification in combination with confocal imaging.

### Functionalization of SWCNTs

1-Pyrenebutanoic acid succinimidyl ester (PASE) was purchased from Life technologies, Singapore. Dimethylformamide (DMF), 100 μM single-stranded probe DNA (5′-NH_2_ (CH_2_)_3_-CAAACACCATTGTCACACTCCA-3′), 100 μM single-stranded complementary DNA (cDNA, 5′-TGGAGTGTGACAATGGTGTTTG-3′), single-stranded non-complementary DNA (ncDNA, 5′-TGG**T**GTGTGACA**G**TGGTGT**A**TG-3′), ethanolamine (EA, pH 9.0) and 0.1% Tween 20 were purchased from Sigma-Aldrich, Singapore. Phosphate buffer saline (PBS, pH 7.4, Biotechnology grade) consisting of 137 mM NaCl, 2.7 mM KCl, and 10 mM phosphate buffer in 1000 mL distilled H_2_O was purchased from BST Scientific Ltd., Singapore.

SWCNT-based FETs were incubated with 5 mM of the linker molecule PASE in pure DMF for 3 hours at room temperature followed by washing with DMF and deionized (DI) water. 5 µL of the 10 µM probe DNA dissolved in PBS was pipetted onto the devices and incubated for 16–24 hours in a humid environment. This was followed by rinsing with PBS and DI water and air drying with nitrogen to remove the unbound probe DNA. Then the devices were incubated with 0.1 M EA for 1 hour to quench the unreacted succinimidyl ester group on the linker molecules. A similar washing procedure was adopted as stated earlier. To passivate the uncoated CNT area, the devices were incubated for 1 hour with 0.1% Tween 20 followed by the same washing procedure. Finally, 5 µL of 1 µM cDNA dissolved in PBS was added to the probe DNA immobilized channel and junction area, and incubated in a humid environment for hybridization to take place. After approximately one hour, the devices were washed with PBS and DI water, and dried in a nitrogen stream. As a control, the devices where only the channel was exposed were tested with 5 µL of cDNA and 1 µM ncDNA.

### Electrical characterization

All electrical measurements were recorded using an Agilent 4156B semiconductor device analyzer under ambient laboratory conditions (25 ºC and <70% humidity). The drain current *(I*_D_) versus gate voltage (*V*_G_) characteristics were measured at a constant drain–source voltage (*V*_DS_) of 1 V. The slope of the *I*_D_*–V*_DS_ curve in the range of −0.1 to 0.1 V at *V*_G_ = −24 V (on state) for the FET gives the on state conductance (*G*_on_). The threshold voltages for the forward (*V*_fth_, −24 to 24 V) and reverse (*V*_rth_, 24 to −24 V) gate voltage sweeps were extracted by extrapolating the steepest portions of the *I*_D_*–V*_DS_ curve for both the gate voltage sweeps to intersect with the x-axis. The instantaneous slope of the *I*_D_*–V*_DS_ curve at a particular *V*_G_ gives the transconductance (*g*_m_) at that *V*_G_. The *g*_m_ of the *I*_D_*–V*_DS_ curve near the threshold voltage gives the peak transconductance (*g*_mp_). The difference between the threshold voltages obtained from the forward and reverse gate voltage sweeps gives the hysteresis gap (*H*). At least 5 devices of each length (*L* = 6 µm, 300 µm and 1500 µm) were fabricated and used for the sensing experiments.

### Calculations

The effective mobility (µ_e_) represents the intrinsic physical charge carrier mobility of the CNT channel without including any device attributes and is given by,

[1]
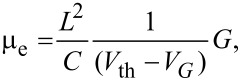


where *G* = δ*I*_D_/δ*V*_DS_ at constant *V*_G_, *G* is the conductance at a particular gate voltage and *V*_th_ is the threshold voltage [24]. The capacitance per unit length of the nanotube, (when modeled as a cylinder on a planar substrate) is given by

[2]
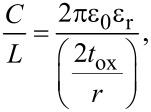


where ε_0_ is the permittivity of free space (8.8542 × 10^−12^ Fm^−1^), ε_r_ is the dielectric constant of the SiO_2_ gate insulator (≈3.9), *t*_ox_ is the SiO_2_ thickness, *r* is the radius of the nanotube, and *L* is the length of the CNT [20,24]. Another definition of mobility, which includes device attributes such as contact resistance, parasitic resistance, and surface effects, is the conventional device field-effect transistor mobility (µ_f_). It is obtained from the *I*_D_*–V*_DS_ curve and is related to *g*_m_ by,

[3]
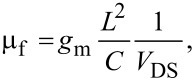


where *g*_m_*=* δ*I*_D_/δ*V*_G_ at a constant *V*_DS_ [20,24]. The influence of various device attributes makes the estimated field-effect mobility different from the effective intrinsic mobility. Therefore, mathematically, µ_f_ can be related to µ_e_ by a device factor constant, φ, that is, µ_f_ = µ_e_/φ, where 1/φ indicates the contribution of device attributes [25–26].

If Δ represents the hybridization-induced, absolute change in mobility, and b and a refer to before hybridization and after hybridization, respectively, then Δµ_e_ and Δµ_f_ can then be expressed in terms of the device factor using µ_eb_ = φ_b_µ_fb_ and µ_ea_ = φ_a_µ_fa_ as,

[4]
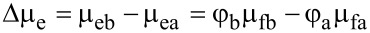


and

[5]
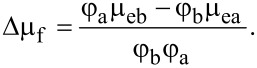


To include the change in *G*, *g*_m_ and *V*_th_ induced by DNA hybridization, and to represent them by a single parameter, we divide Equation 4 by Equation 5:

[6]
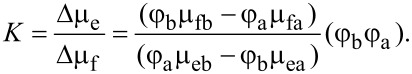


Equation 6 succinctly describes the change in the electrical parameters and is related to φ. If φ remains unaffected after hybridization, then φ_a_ = φ_b_ = φ in Equation 6, resulting in *K* = φ. Hence, Δφ = 0 is the condition for which the contribution of device attributes to mobility change is minimum, that is, *K* = φ will yield a maximum response from the CNT channel. *K* can be used for the analysis of the effect of gate voltage, channel length, channel number, etc. on the response of FET when multiple sensing mechanisms contribute to a change in *G*, *g*_m_ and *V*_th_.

## Results and Discussion

**Figure 1:**
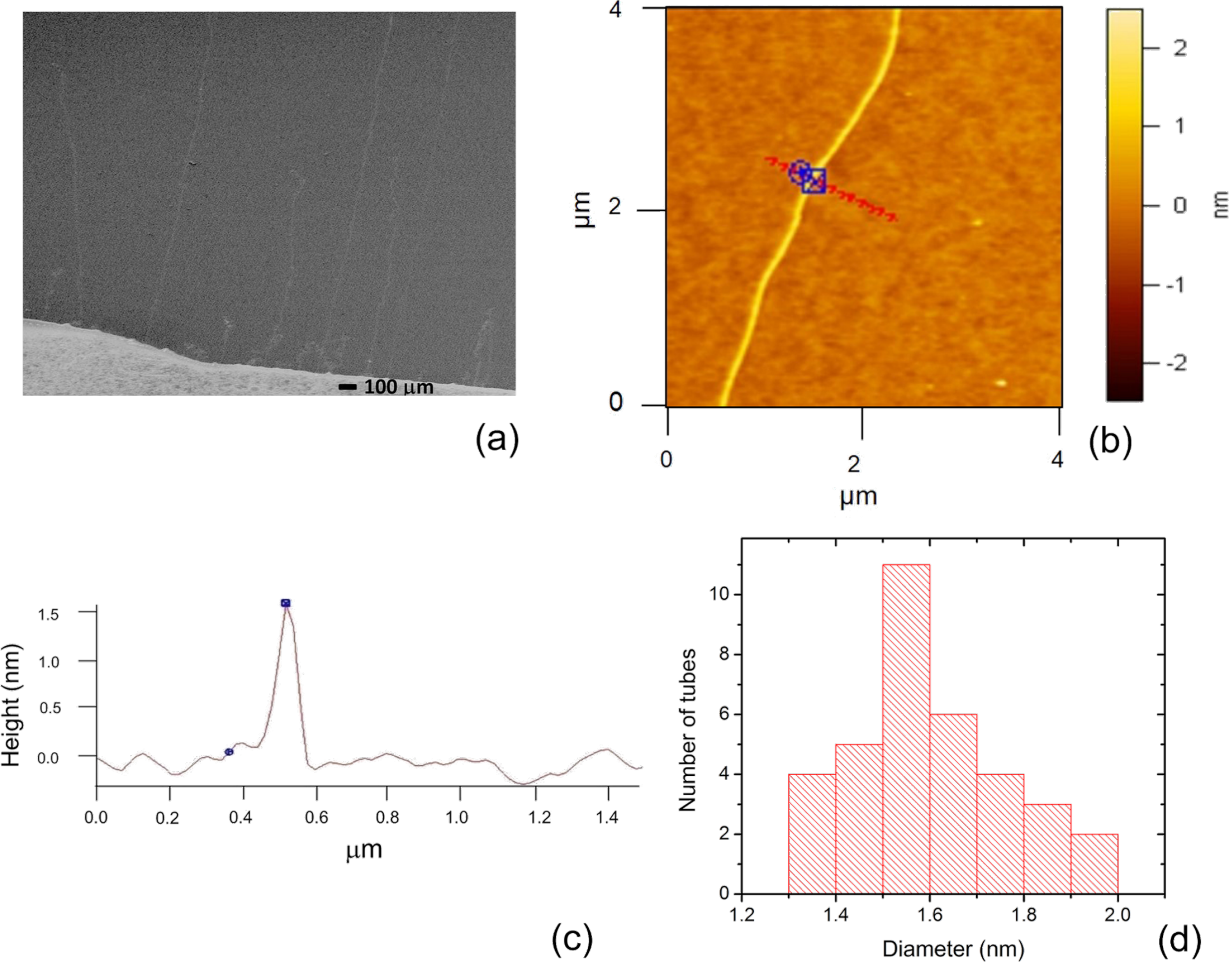
(a) SEM image of long CNTs grown by the CVD process. (b) AFM image and (c) height profile of a long individual SWCNT with a diameter of ≈1.51 nm. (d) A histogram plot of the diameter distribution of the as-grown SWCNTs.

Figure 1a shows an SEM image of the as-grown, long CNTs. Figure 1b,c shows the AFM image and height profile of an individual SWCNT. The diameter distribution of the SWCNTs is shown in Figure 1d. The average diameter of the SWCNTs was ≈1.55 nm.

**Figure 2:**
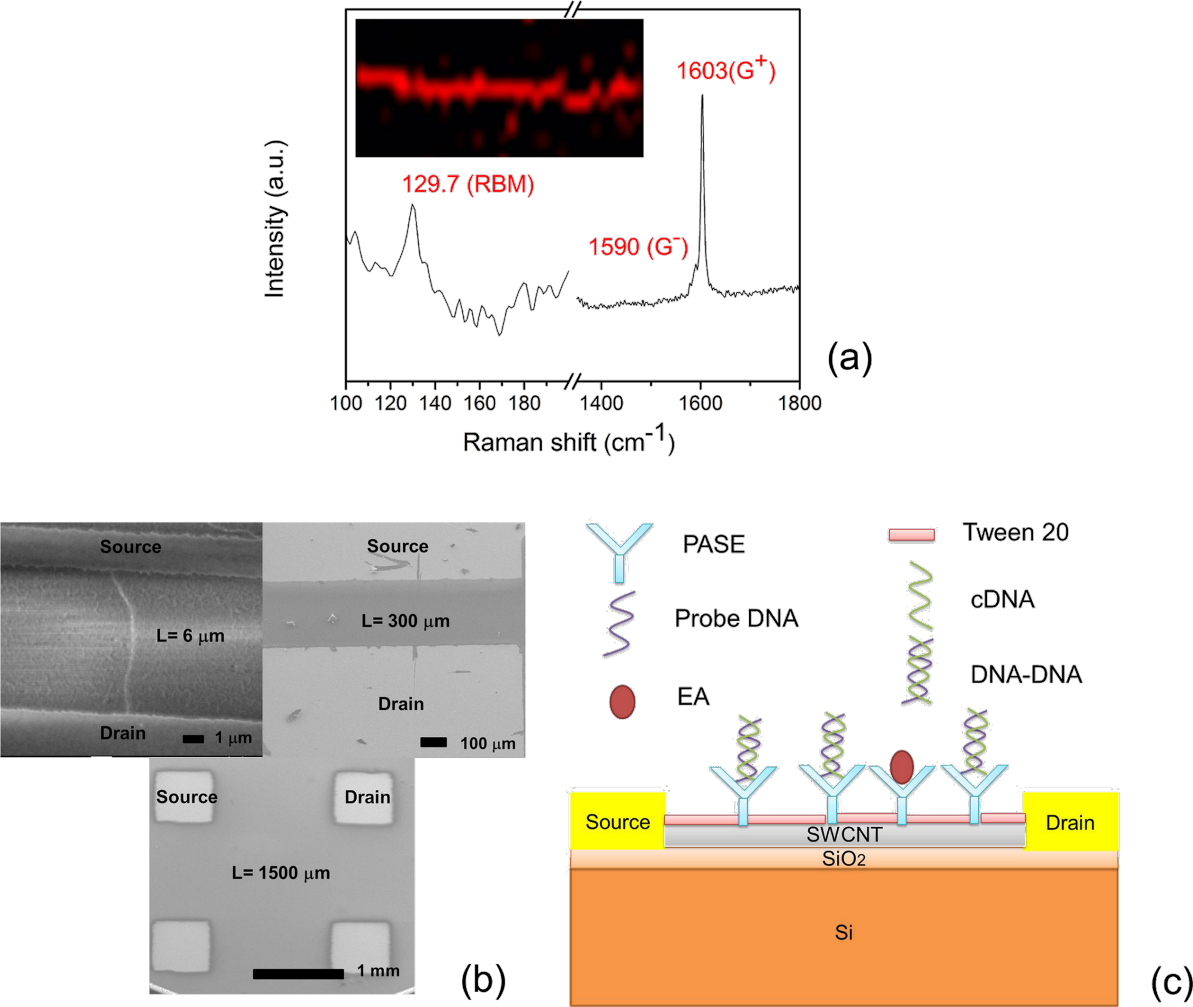
(a) The Raman spectrum of a 1 cm long SWCNT with a RBM at ≈129.7 cm^−1^ and a G-band with G^−^ and G^+^ peaks at 1590 cm^−1^ and 1603 cm^−1^, respectively, under 633 nm wavelength excitation. The inset shows the Raman mapping image of the SWCNT. (b) SEM image of as-prepared SWCNT-based FETs with channel lengths of 6 µm (short), 300 µm (long) and 1500 µm (ultra-long), shown with source and drain electrodes. (c) Schematic setup for DNA hybridization detection using SWCNT-based FET. cDNA represents the complementary target single stranded DNA and DNA–DNA represents the double stranded DNA formed by complementary base pairing between probe DNA and cDNA.

Figure 2a shows a typical Raman spectrum of an individual SWCNT spatially resolved from the Raman mapping image (shown in the inset). A SWCNT with a RBM frequency at 129.7 cm^−1^ is assumed to be semiconducting within the resonance window. The G-band displays a typical Lorentzian line shape with two peaks at 1590 cm^−1^ and 1603 cm^−1^ for G^−^ and G^+^, respectively, which further demonstrated the semiconducting characteristic of the SWCNT.

Figure 2b shows the as-fabricated short, long and ultra-long type of FETs. All of the devices with a particular *L* used here for testing had similar *I*_D_–*V*_G_ characteristics. For *L* = 6 µm, *G*_on , _*g*_mp_, *V*_fth, _*V*_rth_ and *H* were calculated to be 4.5 ± 1.1 × 10^−6^ Ω^−1^, 3.1 ± 1.2 × 10^−6^ Ω^−1^, 0 ± 2 V, 5 ± 2 V and 6 ± 3 V, respectively. For *L* = 300 µm, *G*_on , _*g*_mp_, *V*_fth, _*V*_rth_ and *H* were estimated to be 8.8 ± 1.4 × 10^−8^ Ω^−1^, 6.1 ± 2.4 × 10^−8^ Ω^−1^, −8 ± 2 V, 0 ± 2 V and 10 ± 2 V, respectively. For *L* = 1000 µm, *G*_on , _*g*_mp_, *V*_fth, _*V*_rth_ and *H* were calculated to be 1.4 ± 0.4 × 10^−8^ Ω^−1^, 9 ± 4 ×10^−9^ Ω^−1^, −12 ± 2 V, −2 ± 2 V and 15 ± 3 V, respectively. After the additional photolithographic step for junction capping, *G*_on, _*g*_mp_, *V*_fth, _*V*_rth_ and *H* showed notable changes for the different channel lengths: −16%, −11%, −0.5 V, +0.5 V and +1 V (*L* = 6 µm); −11%, −8%, −1 V, +1 V and +2 V (*L* = 300 µm); and −12%, −10%, −2 V, +2 V and +3 V (*L* = 1000 µm). Here, +/− indicates increase/decrease in *G*_on_, *g*_mp_ and *H,* and a positive/negative shift for *V*_fth_ and *V*_rth_ after the photolithographic step for junction capping. Figure 2c shows a schematic setup of the SWCNT-based FET used to detect DNA hybridization.

We first take the long channel type FET for example to study the response of FET to hybridization. The long channel FETs used in this study exhibited high µ_e_ and µ_f_ values of ≈1.04 × 10^4^ cm^2^ V^−1^ s^−1^ and 2.53 × 10^3^ cm^2^ V^−1^ s^−1^, respectively, obtained using Equations 1 and 3. The output (*I*_D_*–V*_DS_) and transfer (*I*_D_–*V*_G_) curves of the various functionalization steps involved in DNA hybridization detection are given in Figure S1a,b in the Supporting Information File 1. The pyrene group of PASE non-covalently interacted with the CNT through π-stacking forces and the other loose end containing the succinimidyl ester group (or *N*-hydroxysuccinimide, NHS) covalently bound with the highly reactive amine group in the probe DNA. It was observed that the pyrene-functionalized SWCNT-based FETs showed a 5–10% reduction in *I_D_* in the on state and a negative shift in the *V*_fth_ by about −3 V after probe DNA immobilization. This is perceived to be the result of electron doping by negatively charged probe DNA. After addition of EA and Tween 20, a slight reduction in the on current occurred because of carrier scattering by coating of these molecules on the CNT surface.

**Figure 3:**
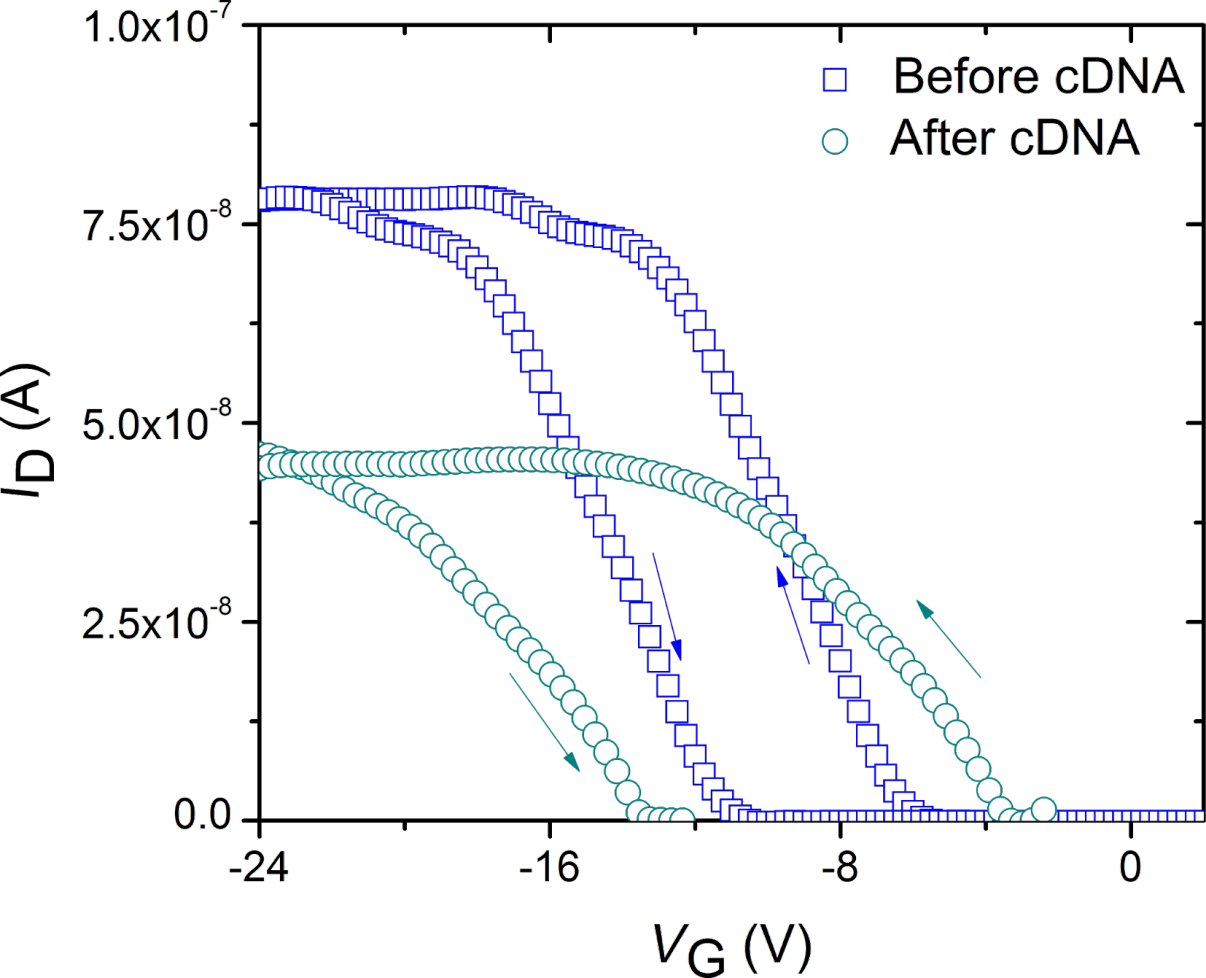
*I*_D_–*V*_G_ curves of SWCNT-based FETs with the channel and junction exposed to cDNA (*L* = 300 µm). The “before cDNA” step corresponds to the recording obtained after PASE, probe DNA, EA and Tween 20 incubation steps. The “after cDNA” step represents the recording after incubation with cDNA. The arrows indicate the direction of the gate voltage sweep.

Figure 3 shows typical *I*_D_–*V*_G_ curves of a SWCNT-based FET (*L* = 300 µm) with the channel and junction exposed to cDNA. After DNA hybridization, *G*_on_ and *g*_mp_ decreased by about 44.4% and 72.3%, respectively. In addition, *V*_fth_ and *V*_rth_ shifted by about −2 V and +2 V, respectively, in the transfer curve resulting in an increase in *H* by about 4 V.

The drop in conductance and transconductance suggests carrier scattering due to the formation of hybridized double stranded DNAs (ds-DNAs) by complementary base pairing between probe DNA and cDNA whereas the negative shift in *V*_fth_ implies electron transfer to the SWCNT by the negatively charged cDNA molecules as the possible mechanisms [27]. The increase in *H* is proportional to the number of charge traps formed by ds-DNA hybrids immobilized on SWCNT, which indicates the contribution of charge-trapping mechanism to the observed behavior [27–28]. Similar DNA detection studies using CNTs based on the principle of impedance exhibited detection limits as low as 100 aM [29].

To verify whether the source of the signal is from the CNT channel, *I*_D_–*V*_G_ curves of FETs with only the channel region exposed to cDNA were analyzed. Such FETs showed similar characteristics to those devices with both the channel and junction exposed to cDNA, that is, a reduced *G*_on_, negative shift in *V*_fth_, decrease in *g*_mp_ and an increase in *H* (Figure S2a in Supporting Information File 1), which suggests that the channel region contributes significantly to the observed response.

Furthermore, to confirm the specificity of complimentary target hybridization, we repeated the experiment using ncDNA for FETs with only the channel exposed, similar to earlier studies [30]. The results show no significant change in the transfer characteristics (Figure S2b in Supporting Information File 1). This suggests that ncDNA failed to induce any response, and the source of signal for the earlier experiment using cDNA is a result of complementary base pairing, and not due to non-specific binding or any other irrelevant biological events.

### The effect of channel length on FET properties after DNA hybridization

After comparing the FET with only the channel exposed to cDNA/ncDNA to those with both channel and junction exposed, we further investigated the effects of *L* on FET properties. Here, the effect of *L* on the change in each of the electrical parameters induced by DNA hybridization is studied by comparing FETs containing three channel lengths: short (*L* = 6 µm), long (*L* = 300 µm) and ultra-long (*L* = 1500 µm) channels.

**Figure 4:**
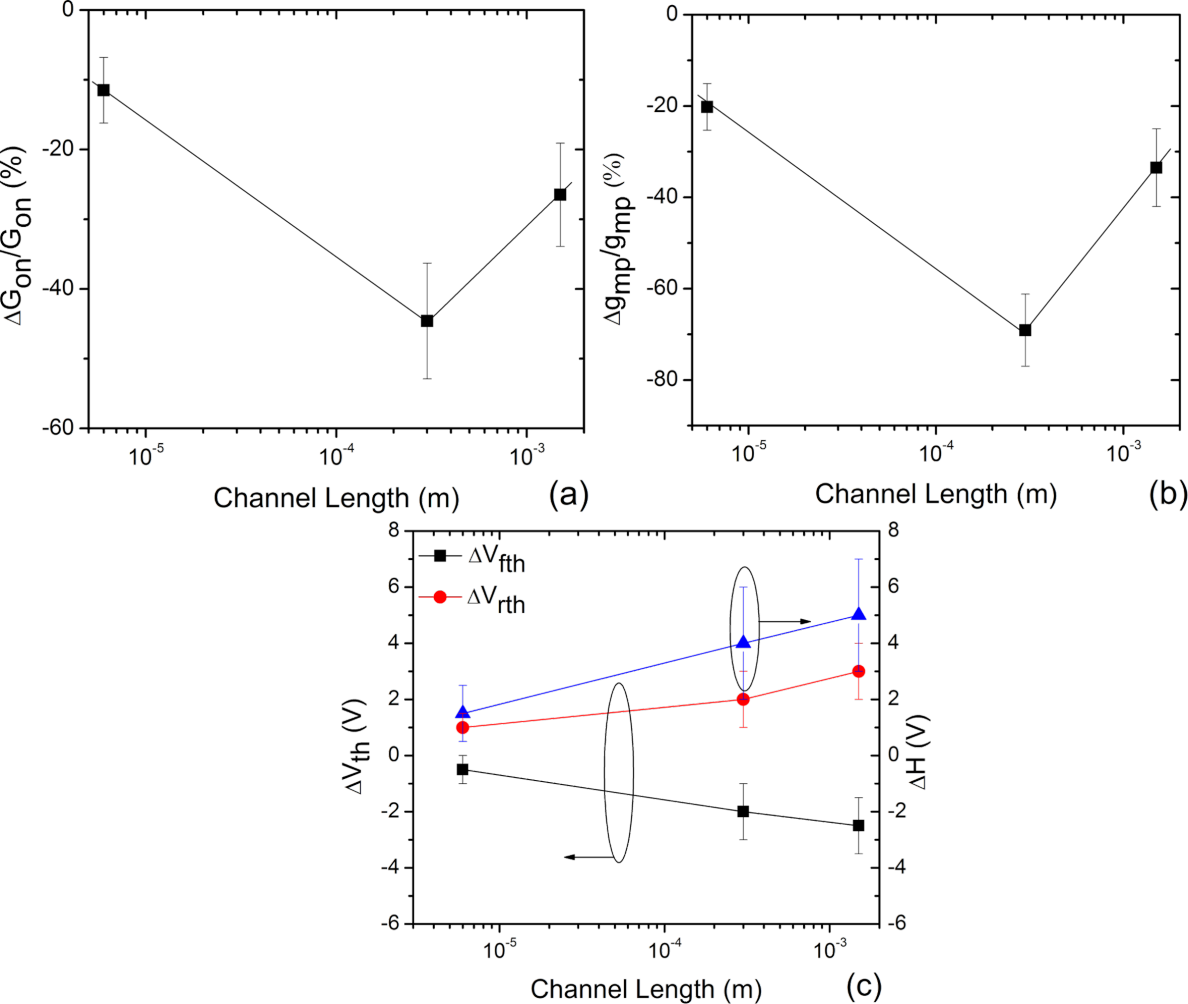
The relative (a) on state conductance (Δ*G*_on_/*G*_on_) and (b) peak transconductance (Δ*g*_mp_/*g*_mp_) after cDNA exposure, expressed in percent, for three different FET channel lengths (*L* = 6 µm, 300 µm and 1500 µm) with both the channel and junction exposed. The negative sign on the y-axis indicates the decrease in magnitude of these two parameters after DNA hybridization. (c) The variation of the change in forward sweep threshold voltage (Δ*V*_fth_), reverse sweep threshold voltage (Δ*V*_rth_) and hysteresis gap (Δ*H*) induced by DNA hybridization with *L*. The negative and positive signs of the threshold voltage shifts indicate the negative and positive shift in the transfer curves. A positive Δ*H* indicates the rise in hysteresis. The changes in all electrical parameters induced by DNA hybridization are obtained with respect to the “before cDNA” *I*_D_*–V*_G_ curves. The solid lines are a guide to the eye.

As shown in Figure 4a, the variation of the relative on state conductance (Δ*G*_on_/*G*_on_) increased from 12% to 47% with increased *L* from 6 to 300 µm, and then decreased to 27% for *L* = 1500 µm. For the same variation in *L*, the relative peak transconductance (Δ*g*_mp_/*g*_mp_) increased from about 20% to 69%, and thereafter, dropped to about 34% as shown in Figure 4b. From Figure 4c, the variation in threshold voltage shift for forward (Δ*V*_fth_) and reverse (Δ*V*_rth_) gate voltage sweeps, and the change in hysteresis gap (Δ*H*) with *L* increased from about −0.5 to −2.5 V, 1 to 3 V and 1.5 to 5 V, respectively. The shift in *V*_th_ to more negative values for the forward sweep indicates electron trapping by the guanine and adenine bases [28,31]. Similarly, the shift in *V*_th_ to more positive values for the reverse sweep is an indicator of hole trapping by the cytosine and thymine bases [28,31]. Thus, the increase in charge traps in the channel region leads to an overall shift in *V*_th_ for forward and reverse sweeps reflected as an increase in hysteresis. This differs from the CNT-FET-based DNA detection studies reported by Gui et al. [32], where charge density modification near the CNT–metal electrode junction through electron doping causes a negative shift in the forward and reverse sweep threshold voltages.

For short channel lengths, the change in contact resistance (Δ*R*_c_) and channel resistance (Δ*R*_ch_) (Figure S3 in Supporting Information File 1 for *L* = 6 µm) contributed significantly to the change in total on state resistance (Δ*R*_on_) after hybridization. Moreover, the detection response of the channel region is low, which indicates that the modulation in the CNT–metal work function also influences the response, in addition to the aforementioned mechanisms [18]. With increasing *L*, the number of additional charges present on the CNT surface in the form of ds-DNA hybrids increases, thereby Δ*V*_th_ increases. Similarly, the additional number of charge traps formed during hybridization increases along the length of the nanotube, thus, offering more trapping/detrapping sites leading to an increase in Δ*H* with *L*. Hence, ultra-long channel FETs exhibited greater charge transfer and charge trapping mechanisms, but the conductance response was lower when comparing with devices of *L* = 300 µm (Figure S3 in Supporting Information File 1 for *L* = 1500 µm), which suggests that a very high channel resistance could be a limiting factor in ultra-long channel FETs [33]. On the other hand, long channel FETs showed appreciable charge transfer and charge trapping mechanisms comparable to the ultra-long channels with high response from the channel, but with low contribution from the CNT–metal junction (Figure S3 in Supporting Information File 1 for *L* = 300 µm). Overall, the 300 µm long channel FETs make use of their excellent coverage area, freedom from contacts and higher mobility to confine more hybridizations in the channel area, while showing better response compared to short channel FETs. In addition, they do not suffer from the very high channel resistance hindering the detection sensitivity compared to ultra-long channel FETs.

Clearly, for all devices, the transfer characteristics showed a change in transconductance, threshold voltage and hysteresis gap, which confirms the contribution of carrier scattering, charge transfer, and charge trapping in detecting hybridization. However, the response had a varying degree of contribution of each mechanism for different *L*, thereby resulting in the observed trend with *L.* These mechanisms include the effect of electron transfer, CNT contact work function modulation, DNA–DNA duplex affinity on the SWCNT, hopping conduction, etc. on the response [27–28,33]. Therefore, it is necessary to consider the contribution of the change in *g*_m_, *V*_th_ and *H* caused by hybridization when explaining this result. Therefore, it is proper to use Δφ and *K* to explain the observed trend in the conductance response.

### Δφ and *K* variation with channel length

**Figure 5:**
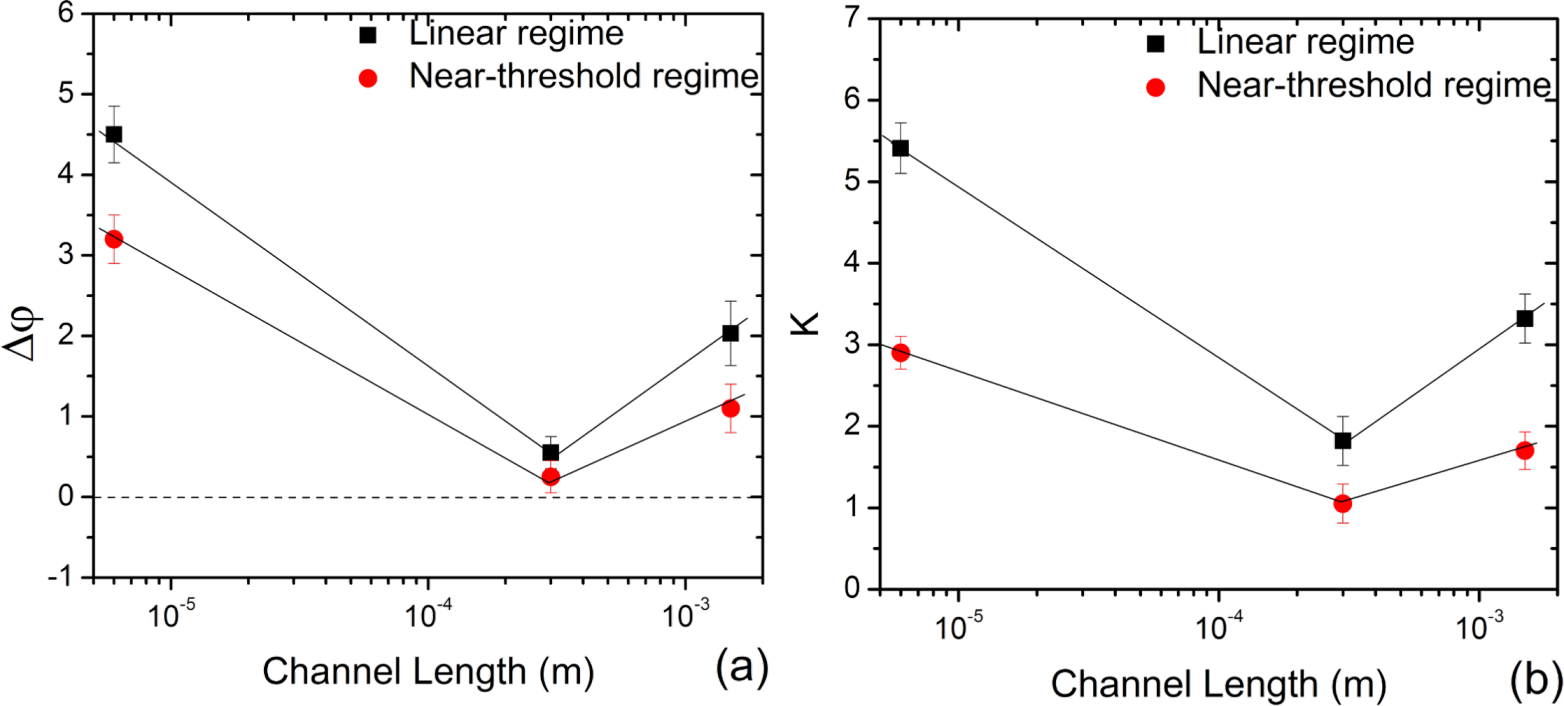
The variation in (a) Δφ and (b) *K* with *L* in the linear and near-threshold regimes for the forward sweep. The solid lines are a guide to the eye.

Depending on the gate bias, Δφ and *K* can be extracted in the linear, near-threshold, or the sub-threshold regimes [34]. Equations 1 and 3 are valid only above the threshold voltage, therefore, we restrict our analysis to the linear (on state) and near-threshold (*g*_mp_) regimes [25]. The variations in Δφ and *K* (forward sweep) with *L* are shown in Figure 5a,b for both linear and near-threshold regimes. We found that with an increase in *L* from 6 µm to 300 µm to 1500 µm, in the linear regime, Δφ decreased from about 5 to 0.6, and then increases to about 2. *K* also follows the same trend and decreases from about 5.4 to 2, and then increases to about 3.3. In the near-threshold regime, Δφ decreases from about 3.2 to 0.3, and then increases to about 1. Furthermore, in this regime, *K* values for the three channel lengths (6 µm, 300 µm and 1500 µm) were calculated to be approximately 3, 1 and 2, respectively. We also estimated Δφ and *K* for the three channel lengths for reverse gate voltage sweep (Figure S4a,b in Supporting Information File 1). The magnitudes of Δφ and *K* were slightly different for forward and reverse sweeps, but the variation trends with *L* remained the same for both regimes.

From the above results, two important points are noteworthy. Firstly, in both of the regimes, Δφ and *K* showed high values for devices with *L* = 6 µm and 1500 µm (Δφ >> 0 and hence *K* ≠ φ), comparing to devices with *L* = 300 µm (Δφ → 0 and hence *K* → φ). Secondly, the magnitude of Δφ and *K* in the linear regime is greater than that in the near-threshold regime. The plausible reasons for the observed behavior are discussed subsequently.

Ideally, Δφ should be close to 0, however, in reality, φ could be influenced by contact resistance [35–36], optical/acoustic phonon scattering [33], and trapped charges [37] depending on *L* and *V*_G_, which leads to Δφ *≠* 0, and consequently, *K ≠* φ. For short channel lengths, after hybridization, Δ*R*_c_ also contributed significantly to Δ*R*_on_, and the CNT–metal work function is altered, which affects the mobility. Phonon scattering is prominent for all *V*_G_ and increases with *L* [38]. The degrading effect of phonon scattering on the mobility with *L* will have a profound impact on the detection capability of the FETs. Finally, the presence of defects at the CNT–gate dielectric interface (interface defects) or within the gate dielectric (bulk defects) results in injected electron trapping/detrapping around the CNT/SiO_2_ surface during forward and reverse gate voltage sweeps, which is responsible for the hysteresis behavior [39]. These charge traps could participate in Coulombic scattering [40], and thereby influence mobility. If Δφ_c_, Δφ_s_ and Δφ_h_ represent the influence of contact resistance, phonon scattering, and charge traps respectively on Δφ, then, the absolute change in the device factor after hybridization (Δφ) is Δφ_c_ + Δφ_s_ + Δφ_h_, which upon on substituting in Equation 6 yields,

[7]
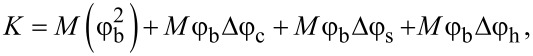


where 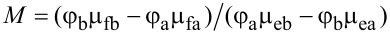.

The influence of the change in contact resistance (i.e., Δφ_c_) reduced with increasing *L*, and conversely, the influence of phonon scattering [38] (i.e., Δφ_s_) reduces with decreasing *L*. If we consider Δφ_c_ >> Δφ_s_ for *L* = 6 µm, Δφ_c_ + Δφ_s_ ≈ 0 for *L* = 300 µm and Δφ_c_ << Δφ_s_ for *L* = 1500 µm, then these devices have an additional term of Δφ_c_ for *L* = 300 µm and Δφ_s_ for *L* = 300 µm (both regimes) resulting in *K* > φ. However, for devices with *L* = 300 µm, Δφ → 0 (both regimes) thereby resulting in *K* → φ, which is in agreement with the first observation.

To illustrate the second observation, we use terms with additional subscripts of “l” and “t” to denote parameters extracted in the linear and near-threshold regimes respectively. In the linear regime (high gate voltage), the influence of Schottky barrier reduces, which results in lower contact resistance [35], as compared to the near-threshold regime (lower gate bias). Therefore, the contribution from the contacts to the mobility change at higher gate voltage is greater compared to lower gate voltage, that is, φ_cl_ >> φ_ct_. Moreover, phonon scattering exists at all *V*_G_ [38], therefore, φ_cl_ + φ_sl_ >> φ_ct_ + φ_st_. The influence of φ_hl_ is neglected in the linear regime because the potential of the back gate is high enough to convert all injected charges in SiO_2_ into holes, that is, in this regime all the charge traps are in the filled state [35,37]. On the contrary, in the near-threshold regime, the lack of dissipation of injected charges on account of hysteresis causes the potential induced by the trapped charges to exceed the potential of the back gate voltage [37], which emphasized the need to include the influence of trapped charges (Δφ_h_) in Δφ. However, earlier reports [40] suggested that Coulombic scattering by trapped charges have a weaker effect on the CNT mobility than acoustic phonon scattering at lower gate voltage, that is, φ_st_ >> φ_ht_. Thus, taking into consideration all these factors, the magnitudes of Δφ and *K* in the linear regime are greater when comparing to those obtained in the near-threshold region (Δφ_l_ > Δφ_t_ and hence *K*_l_ > *K*_t_). These results suggest that the near-threshold regime will produce greater response to hybridization with minimum variation in device attributes as compared to the linear regime for all channel lengths.

The variation of Δφ and *K* with *L* for both the regimes is similar to the trend observed in the variation of Δ*G*_on_/*G*_on_ with *L.* Evidently, from the Δφ versus *L* and *K* versus *L* plots (Figure 5a,b and Figure S4a,b in Supporting Information File 1) when compared to the other channel lengths, the FETs with *L* = 300 µm approach the condition of Δφ = 0, and hence *K* = φ, thereby resulting in the highest response to DNA hybridization.

## Conclusion

DNA hybridization was detected using individual SWCNT-based FETs with different channel lengths. The SWCNT-based FET for hybridization detection resulted in a reduced conductance and transconductance, a shift in threshold voltage, and an increase in the hysteresis gap, which varied with channel length. The response of FETs employing 300 µm long SWCNTs exhibited the best response to hybridization, when compared to short and ultra-long channels. Using the defined parameters Δφ and *K*, which include the change caused by hybridization in the different electrical FET parameters, it was found that such long channel FETs showed the least variation in device factor after hybridization, namely that Δφ → 0 and hence *K →* φ, resulting in the maximum response to hybridization.

## Supporting Information

The Supporting Information contains 1) *I*_D_–*V*_DS_ and *I*_D_–*V*_G_ plots of different functionalization steps, 2) *I*_D_–*V*_G_ plots comparing the response to cDNA and ncDNA in channel-exposed FETs, 3) the variation in the conductance response of channel and junction exposed devices with *L* and 4) plots of Δφ and *K* variation with *L* in the linear and near-threshold regimes for the reverse sweep.

File 1 Additional experimental data.

## References

[1] Li H, LaBean T H, Leong K W (2011). Interface Focus.

[2] Mothershed E A, Whitney A M (2006). Clin Chim Acta.

[3] Weiss F U, Marques I J, Woltering J M, Vlecken D H, Aghdassi A, Partecke L I, Heidecke C-D, Lerch M M, Bagowski C P (2009). Gastroenterology.

[4] Pena J T G, Sohn-Lee C, Rouhanifard S H, Ludwig J, Hafner M, Mihailovic A, Lim C, Holoch D, Berninger P, Zavolan M (2009). Nat Methods.

[5] Siva A C, Nelson L J, Fleischer C L, Majlessi M, Becker M M, Vessella R L, Reynolds M A (2009). Mol Cancer.

[6] Liang R-Q, Li W, Li Y, Tan C-y, Li J-X, Jin Y-X, Ruan K-C (2005). Nucleic Acids Res.

[7] Tang Y, Achyuthan K E, Whitten D G (2009). Langmuir.

[8] Gouttenoire V, Barois T, Perisanu S, Leclercq J-L, Purcell S T, Vincent P, Ayari A (2010). Small.

[9] Kang S J, Kocabas C, Ozel T, Shim M, Pimparkar N, Alam M A, Rotkin S V, Rogers J A (2007). Nat Nanotechnol.

[10] Chen C-L, Yang C-F, Agarwal V, Kim T, Sonkusale S, Busnaina A, Chen M, Dokmeci M R (2010). Nanotechnology.

[11] Khamis S M, Jones R A, Johnson A T C, Preti G, Kwak J, Gelperin A (2012). AIP Adv.

[12] Oh J, Yoo G, Chang Y W, Kim H J, Jose J, Kim E, Pyun J-C, Yoo K-H (2013). Biosens Bioelectron.

[13] Palaniappan A, Goh W-H, Tey J N, Wijaya I P M, Moochhala S M, Liedberg B, Mhaisalkar S G (2010). Biosens Bioelectron.

[14] Feigel I M, Vedala H, Star A (2011). J Mater Chem.

[15] Okuda S, Okamoto S, Ohno Y, Maehashi K, Inoue K, Matsumoto K (2012). J Phys Chem C.

[16] Fu D, Okimoto H, Lee C, Takenobu T, Iwasa Y, Kataura H, Li L-J (2010). Adv Mater.

[17] Sorgenfrei S, Chiu C-y, Gonzalez R L, Yu Y-J, Kim P, Nuckolls C, Shepard K L (2011). Nat Nanotechnol.

[18] Byon H R, Choi H C (2006). J Am Chem Soc.

[19] Heller I, Janssens A M, Männik J, Minot E D, Lemay S G, Dekker C (2007). Nano Lett.

[20] Dürkop T, Getty S A, Cobas E, Fuhrer M S (2003). Nano Lett.

[21] Liu Z, Jiao L, Yao Y, Xian X, Zhang J (2010). Adv Mater.

[22] An J, Zhan Z, Hari Krishna S V, Zheng L (2014). Chem Eng J.

[23] Zhang Y, Zheng L (2010). Nanoscale.

[24] Khanal D R, Levander A X, Yu K M, Liliental-Weber Z, Walukiewicz W, Grandal J, Sánchez-García M A, Calleja E, Wu J (2011). J Appl Phys.

[25] Krishna S V H, An J, Zheng L (2013). J Nanoelectron Optoelectron.

[26] Servati P, Nathan A, Amaratunga G A J (2006). Phys Rev B.

[27] Martínez M T, Tseng Y-C, Ormategui N, Loinaz I, Eritja R, Bokor J (2009). Nano Lett.

[28] Giese B (2002). Annu Rev Biochem.

[29] Kurkina T, Vlandas A, Ahmad A, Kern K, Balasubramanian K (2011). Angew Chem, Int Ed.

[30] Gui E-L, Li L-J, Lee P S, Lohani A, Mhaisalkar S G, Cao Q, Kang S J, Rogers J A, Tansil N C, Gao Z (2006). Appl Phys Lett.

[31] Kan Y (1999). J Am Chem Soc.

[32] Gui E L, Li L-J, Zhang K, Xu Y, Dong X, Ho X, Lee P S, Kasim J, Shen Z X, Rogers J A (2007). J Am Chem Soc.

[33] Guo J, Lundstrom M (2005). Appl Phys Lett.

[34] Gao X P A, Zheng G, Lieber C M (2009). Nano Lett.

[35] Jo G, Maeng J, Kim T-W, Hong W-K, Choi B-S, Lee T (2007). J Appl Phys.

[36] Wang S D, Minari T, Miyadera T, Tsukagoshi K, Tang J X (2009). Appl Phys Lett.

[37] Ong H G, Cheah J W, Zou X, Li B, Cao X H, Tantang H, Li L J, Zhang H, Han G C, Wang J (2011). J Phys D: Appl Phys.

[38] Hamieh S D, Desgreys P, Naviner J F (2010). Eur Phys J B.

[39] Jin S H, Islam A E, Kim T-i, Kim J-h, Alam M A, Rogers J A (2012). Adv Funct Mater.

[40] Estrada D, Dutta S, Liao A, Pop E (2010). Nanotechnology.

