# Supplementary material for: Effect of channel length on the electrical response of carbon nanotube field-effect transistors to deoxyribonucleic acid hybridization
Source: Beilstein J Nanotechnol. 2014 Nov 12;5:2081–91. doi: 10.3762/bjnano.5.217 (PMC4273243; doi:10.3762/bjnano.5.217)
Supplement: File 1 — Additional experimental data. [file Beilstein_J_Nanotechnol-05-2081-s001.pdf]

Supporting Information

for

Effect of channel length on the electrical response of carbon nanotube field-effect transistors to deoxyribonucleic acid hybridization

Hari Krishna Salila Vijayalal Mohan*[1], Jianing An[1], Yani Zhang[2,3], Chee How Wong[1] and Lianxi Zheng*[1,4]

Address: [1]School of Mechanical and Aerospace Engineering, Nanyang Technological University, 50 Nanyang Avenue, 639798, Singapore, [2]Temasek Laboratories, Nanyang Technological University, 50 Nanyang Avenue, 639798, Singapore, [3]Science and Technology on Thermostructural Composite Materials Laboratory, Northwestern Polytechnical University, 710072, Xi'an, PR China, and [4]Department of Mechanical Engineering, Khalifa University of Science, Technology & Research (KUSTAR), P.O. Box 127788, Abu Dhabi, United Arab Emirates

Email: Hari Krishna Salila Vijayalal Mohan* - hari1@e.ntu.edu.sg; Lianxi Zheng* - Lianxi.zheng@kustar.ac.ae

* Corresponding author

Additional experimental data

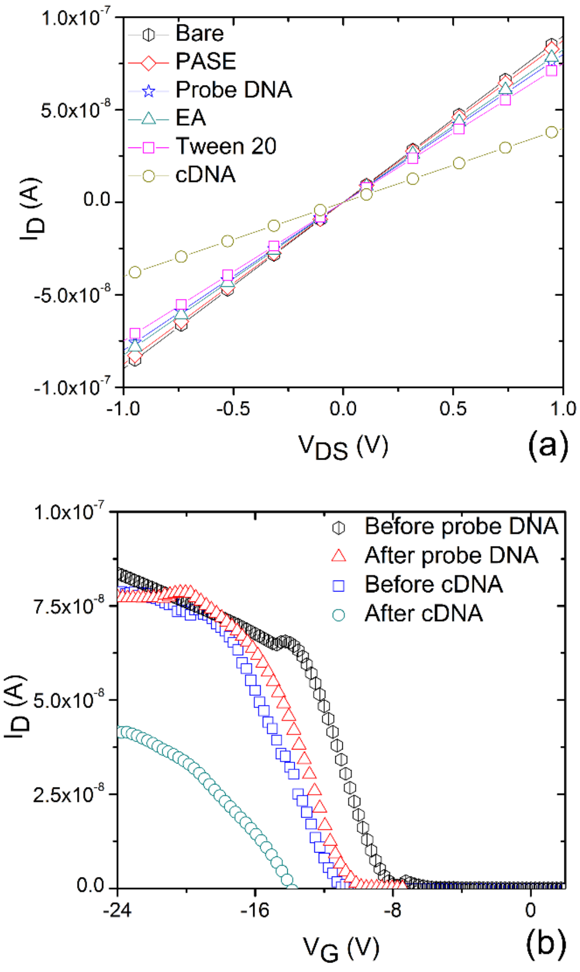

Figure S1: (a) I_D–V_{DS} and (b) I_D–V_G curves of SWCNT-based FETs showing the various functionalization steps with both channel and junction exposed to cDNA (L = 300 µm). The "before probe DNA" and "after probe DNA" I_D–V_G curves represent the PASE functionalization and probe DNA incubation steps, respectively. The "before cDNA" step corresponds to the incubation of probe DNA-immobilized FETs with EA and Tween 20. The "after cDNA" step represents the recording after incubation with cDNA.

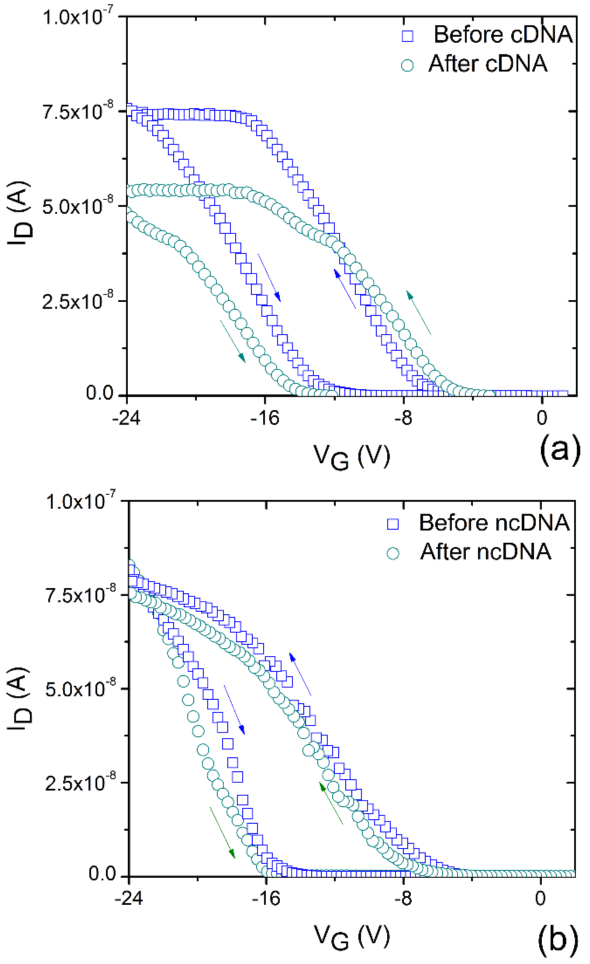

Figure S2: I_D–V_G plots of SWCNT-based FETs (L = 300 μm) with only the channel exposed to (a) cDNA and (b) ncDNA. The "before cDNA" step corresponds to the incubation of probe DNA-immobilized FETs with EA and Tween 20. The "after cDNA" step represents the recording after incubation with cDNA. The arrows indicate the direction of the gate voltage sweep.

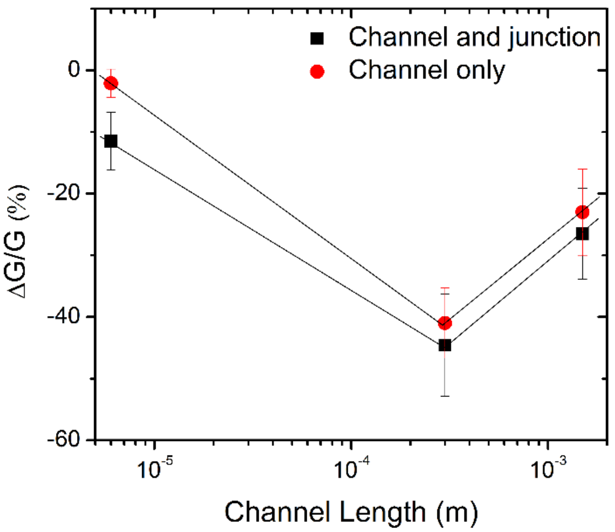

Figure S3: The variation in the conductance response for the devices with the channel and junction exposed ($\Delta G_{on}/G_{on}$) and devices with only the channel exposed ($\Delta G_{ch}/G_{ch}$) to cDNA, both as a function of L. $\Delta G_{ch}/G_{ch} \cong \Delta G_{on}/G_{on}$ for L = 300 µm and 1500 µm, which indicates the dominance of the change in channel resistance (ΔR_{ch}) after hybridization. $\Delta G_{on}/G_{on} > \Delta G_{ch}/G_{ch}$ for L = 6 µm, which indicates the additional contribution of the change in contact resistance (ΔR_c) to $\Delta G_{on}/G_{on}$ after hybridization. The solid lines are a guide to the eye.

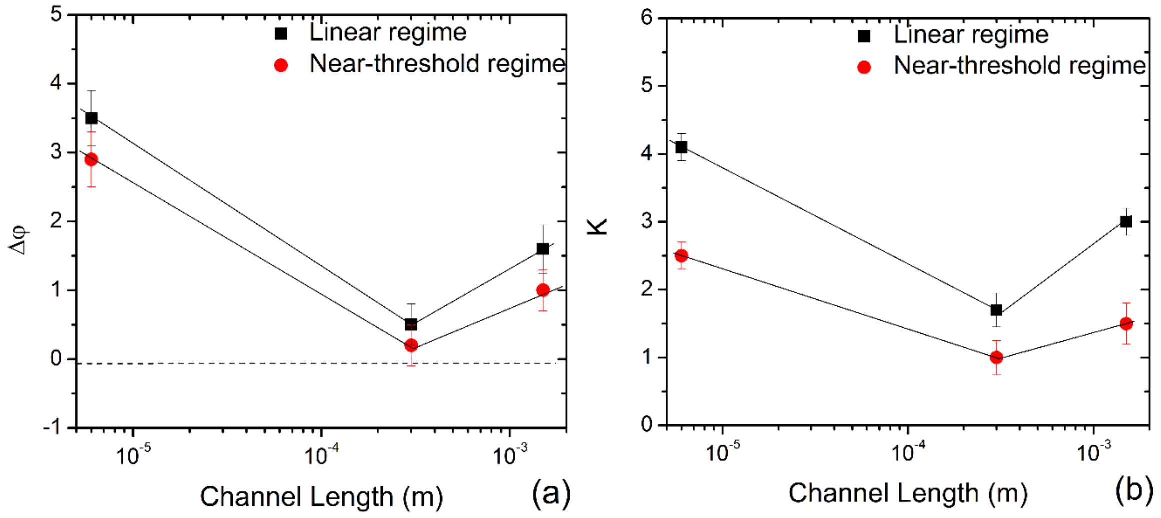

Figure S4: The variation in (a) $\Delta\varphi$ and (b) K with L in the linear and near-threshold regimes for the reverse sweep. The solid lines are a guide to the eye.
